# Added prognostic value of fat attenuation index and CT-derived fractional flow reserve over plaque burden in suspected CAD patients without standard modifiable risk factors

**DOI:** 10.4314/ahs.v24i3.49

**Published:** 2024-09

**Authors:** Yang Yu, Fuqian Guo, Yicheng Chen, Wenjun Bao, Caiying Li

**Affiliations:** 1 Department of Medical Imaging, The Second Hospital of Hebei Medical University, Shijiazhuang, China; 2 Department of Medical Imaging, Cangzhou People's Hospital, Cangzhou, China

**Keywords:** Coronary artery disease, CT-derived fractional flow reserve, fat attenuation index, plaque burden, standard cardiovascular risk factor, prognosis

## Abstract

**Background:**

In this study, we aimed to investigate the added prognostic value of fat attenuation index (FAI) and CT-derived fractional flow (CT-FFR) over plaque burden in suspected coronary artery disease (CAD) patients without standard modifiable risk factors (SMuRFs).

**Methodology:**

A total of 260 consecutive suspected CAD subjects without SMuRFs who underwent first coronary computed tomography angiography (CCTA) were retrospectively collected. We calculated FAI, CT-FFR, and segment involvement score (SIS) from CCTA images. Cox regression models were used to assess the incremental prognostic value of FAI and CT-FFR.

**Results:**

During a median follow-up of 25.00 months, major adverse cardiovascular events (MACE) were observed in 40 (15.4%) patients. FAI ≥ −70.1, CT-FFR ≤ 0.80, and SIS ≥ 4.5 were associated with the increased rate of MACE (*P* < 0.0001). FAI did not provide incremental prognostic value over SIS (*P* = 0.169). Likewise, CT-FFR did not enhance risk prediction (*P* = 0.159). Combining FAI and CT-FFR added incremental prediction value and improved risk discrimination (*P* = 0.032; Absolute integrate discrimination improvement (IDI) = 0.070, *P* < 0.001).

**Conclusion:**

In suspected CAD patients without SMuRFs, neither FAI nor CT-FFR independently added incremental prognostic value over plaque burden. Combining FAI and CT-FFR had added prognostic value and improved cardiovascular risk stratification.

## Introduction

Although cardiovascular medicine has made tremendous progress in the early identification and treatment against standard modifiable risk factors (SMuRFs: smoking, hypertension, diabetes, and hyperlipidemia) for coronary artery disease (CAD), CAD remains the major cause of death worldwide^1^,^2^. Recent studies show that about 15% of patients with ST-segment elevation myocardial infarction do not have SMuRFs [termed SMuRF-less]^3^,^4^.

Notably, the prevalence among SMuRF-less patients has increased from 13% to 27%^5^,^6^. Compared with patients with SMuRFs, SMuRF-less patients remain at an increased risk of recurrent cardiovascular events and death^3^,^7^. Therefore, it is important to further improve the risk stratification of CAD patients without SMuRFs that appropriate management can be provided.

A previous study reported that coronary computed tomography angiography (CCTA) provided an important opportunity for enhancing the stratification of suspected CAD with and without SMuRFs^8^. Another study showed that segment involvement score (SIS) offered added prognostic value over CCTA^9^. However, CCTA provided only anatomic information. Accordingly, fat attenuation index (FAI) and CT-derived fractional flow reserve (CT-FFR) were recently developed as potential non-invasive functional parameters^10^,^11^. More importantly, multiple studies have validated the prognostic performance of FAI and CT-FFR in known or suspected CAD patients^10^,^12^.

Risk stratification only based on a single indicator is oversimple. Given this, several studies have investigated the added predictive value of parameters. In patients referred for CCTA, FAI or CT-FFR provided incremental prognostic value by adding functional information^10^,^13^. Susan et al.^14^ demonstrated that FAI did not provide added prognostic value over myocardial perfusion imaging (MPI) including coronary artery calcium scoring. However, whether FAI and CT-FFR have incremental prognostic value over plaque burden remains elusive. Hence, the present study aims to examine the added prognostic value of FAI and CT-FFR over SIS in suspected CAD patients without SMuRFs.

## Methods

### Study population

**Figure 1:**
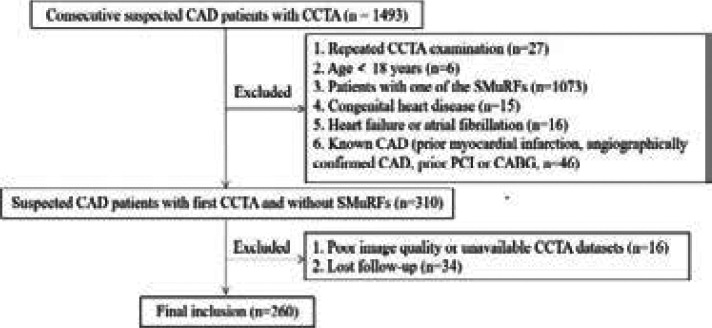
Flow chart. CAD - coronary artery disease; CCTA - coronary computed tomography angiography; CABG - coronary artery bypass graft; PCI - Percutaneous coronary intervention; SMuRFs - Standard modifiable cardiovascular risk factors

This is a single-center retrospective study. From Dec 2019 to Jun 2021, 1493 consecutive individuals who underwent CCTA for evaluation of suspected CAD were initially enrolled. The inclusion criteria were patients aged 18 or older and presented with suspected CAD. The exclusion criteria were as follows: (1) patients younger than 18 years (n=6) and repeated CCTA examination in the database (n=27); (2) patients with one of the SMuRFs (n=1073); (3) patients with congenital heart disease (n = 15); (4) heart failure or atrial fibrillation (n = 16); (5) patients with known CAD (prior myocardial infarction, angiographically confirmed CAD, prior PCI or CABG, n = 46); (6) poor image quality or unavailable CCTA data (n = 16), and (7) lost follow-up (n = 34) (Figure 1). Finally, a total of 260 suspected CAD patients with first CCTA and without SMuRFs were collected in the study. Baseline clinical and imaging data were collected from electronic medical records of the hospital and patient phone calls. This study was approved by the local Ethics Committee.

### Definition of SMuRFs

SMuRFs were defined as current smoking, hyperlipidaemia, hypertension, and diabetes mellitus. Current smoking was defined as regular smoking (≥1 cigarette per day) during the past month before the index hospitalisation. It was considered that the patient had a known history of hypertension, hyperlipidemia or diabetes if they were confirmed to have a history of the same or if they were receiving medication for these conditions at the time of presentation.

### CCTA techniques

CCTA examinations were performed with Phillips 256-slice CT (Brilliance iCT, Philips Healthcare, Amsterdam, Netherlands) and retrospectively gated electrocardiograph (ECG)-triggered spiral data acquisition. Oral metoprolol was given in patients with heart rate ≥ 65 beats/min. Sublingual nitroglycerin was administered to all patients before the examination. During image acquisition, 50–80 mL of iohexol (350 mg/mL, 1.0 mL/kg) was injected at a flow rate of 6–7 mL/s, followed by a 50-ml saline flush. The following scanning parameters were used: 650-1000 mAs tube current, 120 kV tube voltage, 256 × 0.625 mm detector collimation, 0.90 mm layer thickness, 0.30 second/rotation gantry rotation speed.

### FAI, CT-FFR, and SIS measurements

**Figure 2:**
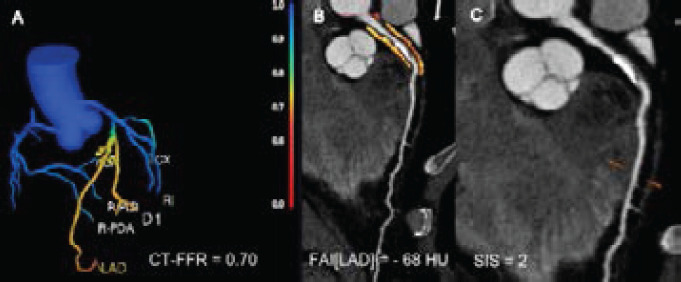
A representative case of CT-FFR, FAI, and SIS assessment. (A) The CT-FFR illustration revealed hemodynamically significant lesion with the CT-FFR value of 0.70. (B) Perivascular FAI phenotyping from CCTA in the proximal 0-40 mm of the LAD. The FAI[LAD] value was -68 HU. (C) CCTA showed atherosclerotic plaques and SIS of 2. CT-FFR - CT-derived fractional flow reserve; CCTA - coronary computed tomography angiography; FAI - fat attenuation index; LAD - left anterior descending artery; SIS - segment involvement score

All images were analysed at the core laboratory by two investigators (7 and 3 years of experience in cardiac imaging diagnosis) independently. The two investigators were blinded to other clinical information. CCTA images were evaluated on Philips iCT EBW 4.5 post-processing workstation (Philips Healthcare, Amsterdam, Netherlands). CT-FFR and FAI were analysed from CCTA images using Deep Learning-based approach software (CT-FFR V1.7, FAI V1.2, ShuKun Technology Co., Ltd., Beijing, China). With this software, CT-FFR values were calculated for each vessel by the deep learning algorithm, 2-4 cm distal to a focal coronary lesion. For the prognostic analysis, the lowest CT-FFR value of each patient was used. The CT-FFR value was provided throughout the coronary arterial tree (Figure 2A). Patients with CT-FFR ≤ 0.80 were categorized as having lesion-specific ischemia^15^. To measure the perivascular FAI, we analysed the proximal 10-50 mm of the right coronary artery (RCA) excluding the first 10 mm, proximal 40 mm of left anterior descending artery (LAD) starting at their origin, and proximal 40 mm of left circumflex (LCX) starting at their origin. The FAI was defined as the mean CT attenuation value of peri-coronary adipose tissue of the traced 40 mm segment by the crude analysis. Representative images of FAI analysis were shown in (Figure 2B). The cutoff point for perivascular FAI was -70.1^10^. SIS was used to quantify burden plaque using CCTA. The SIS (range 1-17) corresponded to the total number of diseased segments, regardless of stenosis severity, and each segment was individually scored as 0 or 1 depending on the presence of plaque (Figure 2C) 16. The sum of all relevant segments for each patient was calculated.

### Follow-up

All study subjects were followed for a median of 25.00 (21.00-30.00) months for outcomes until Aug 31, 2022. The outcomes were major adverse cardiovascular events (MACE), defined as a composite of all-cause death, nonfatal myocardial infarction^17^, late revascularization (more than 90 days after CCTA), or rehospitalization for heart failure. The outcomes were obtained by telephone contact and review of medical records.

### Statistical analysis

Continuous variables were presented as mean ± standard deviation (SD) or medians (IQR). Categorical data were displayed as percentage. The Mann-Whitney U test was used to compare continuous variables, and the Chi-square test or Fisher's exact test to compare categorical variables. Time-to-event was estimated using Kaplan-Meier survival curves and log-rank test. Prognostic value of variables for MACE were performed with univariable and multivariable Cox regression analysis. Any risk factor that had statistically significant (*P* < 0.05) on univariable analysis was selected for multivariable Cox modelling. Relative risks were expressed as multivariable-adjusted hazard ratios (HR) with 95% confidence intervals (CI). The incremental prognostic value of FAI and CT-FFR were assessed by C-index in Multivariable Cox regression models. Furthermore, absolute integrated discrimination improvement (IDI) was used to quantify the risk reclassification probability of models that included FAI and/or CT-FFR to clinical predictors and SIS^18^-^20^,^19^. A 2-tailed P value < 0.05 was considered statistically significant. Statistical analyses were done with R software (version 4.0.2, R Foundation for Statistical Computing, Vienna, Austria) and SPSS software (version 25.0, IBM Corp., Armonk, NY, USA).

## Results

### Patient characteristics

Our study consisted of 260 suspected CAD patients without SMuRFs. Of the 260 patients, the average age was 60.6±9.9 years, and 38.8% (101 of 260) were males. During the median follow-up of 25.00 months, 40 (15.4%) patients experienced at least one MACE. The baseline characteristics of patients are shown in Table 1. In patients with MACE, the rate of males, FAI ≥ -70.1, and CT-FFR ≤ 0.80 were significantly higher compared with patients without MACE. Patients with MACE had lower CT-FFR, whereas patients without MACE had lower FAI[LAD]/[LCX] and SIS.

**Table 1:** Baseline characteristics in patients

Parameters	All n = 260	MACE n = 40	No MACE n = 220	*P*-value
Age, years, mean ± SD	60.6 ± 9.9	61.0 ± 10.8	60.6 ± 9.8	0.889
Sex (male), n (%)	101 (38.8)	24 (60.0)	77 (35.0)	0.003
BMI, kg/m2, mean ± SD	24.9 ± 3.4	24.2 ± 3.1	25.1 ± 3.4	0.129
Family history of CAD, n (%)	6 (2.3)	2 (5.0)	4 (1.8)	0.232
Aspirin/Clopidogrel, n (%)	224 (86.2)	35 (87.5)	189 (85.9)	0.789
Beta-blocker, n (%)	136 (52.3)	24 (60.0)	112 (50.9)	0.290
ACEI, n (%)	6 (2.3)	1 (2.5)	5 (2.3)	1.000
Statin, n (%)	237 (91.2)	36 (90.0)	201 (91.4)	0.763
FAI[LAD], HU, median (IQR)	-84 (-89--78)	-81 (-86--72)	-84 (-90--79)	0.002
FAI[LCX], HU, median (IQR)	-81 (-87--75)	-75 (-83--69)	-81 (-87--76)	0.000
FAI[RCA], HU, median (IQR)	-87 (-93--81)	-86 (-92--79)	-87 (-93--82)	0.280
FAI ≥ -70.1, n (%)	57 (21.9)	20 (50.0)	37 (16.8)	0.000
CT-FFR, median (IQR)	0.89 (0.77-0.93)	0.75 (0.70-0.84)	0.91 (0.81-0.94)	0.000
CT-FFR ≤ 0.80, n (%)	82 (31.5)	29 (72.5)	53 (24.1)	0.000
SIS, median (IQR)	3.0 (2.0-5.0)	5.0 (3.0-7.0)	3.0 (2.0-4.0)	0.000

Data are presented as n (%), mean ± SD or median (interquartile range)

ACEI - angiotensin converting enzyme inhibitor; BMI - body mass index; CAD - coronary artery disease; CT-FFR - CT-derived fractional flow reserve; FAI - fat attenuation index; LAD - left anterior descending artery; LCX - left circumflex; MACE - major adverse cardiac events; RCA - right coronary artery; SIS - segment involvement score

### Prognostic value of parameters

ROC analyses revealed that best cut-off value of SIS to predict MACE was 4.5.

**Figure 3:**
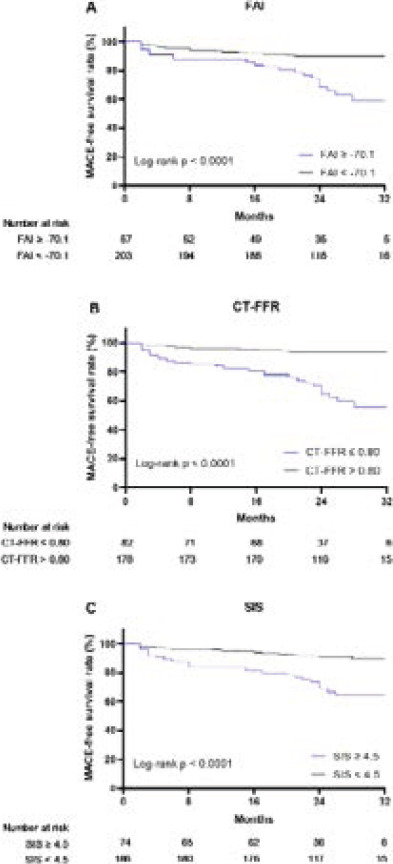
Kaplan-Meier curves for MACE-free rate according to (A) FAI classification; (B) CT-FFR; (C) SIS. CT-FFR - CT-derived fractional flow reserve; FAI - fat attenuation index; SIS - segment involvement score

Kaplan-Meier curves are shown in Figure 3. FAI ≥ −70.1, CT-FFR ≤ 0.80, and SIS ≥ 4.5 were associated with the increased rate of MACE (all log-rank p < 0.0001). Based on univariate Cox regression analysis, sex, FAI ≥ −70.1, CT-FFR ≤ 0.80 and SIS ≥ 4.5 were all significant predictors for MACE (Table 2). Table 3 and 4 summarizes findings of the multivariable-adjusted Cox models. In all models, sex did not have independent predictive value (P > 0.05). In model 1, SIS significantly predicted MACE (HR 3.309, 95% CI 1.727-6.342, P = 0.000). Adding FAI ≥ −70.1 to model 1 did not provide incremental prognostic value (C-index = 0.739, P = 0.169; Absolute IDI 0.012, P = 0.179). Likewise, adding CT-FFR ≤ 0.80 to model 1 did not significantly predict MACE better (C-index = 0.742, P = 0.159; Absolute IDI 0.006, P = 0.261). However, a model with both FAI ≥ −70.1 and CT-FFR ≤ 0.80 had better prediction value than model 1 (C-index = 0.806, P = 0.032; Absolute IDI 0.070, P < 0.001).

**Table 2:** Univariate analysis for MACE in suspect CAD patients without SMuRFs

	HR (95% CI)	*P* value
Age	1.004 (0.972-1.037)	0.810
Sex (Male)	2.555 (1.356-4.815)	0.004
BMI	0.937 (0.852-1.031)	0.182
Family history of CAD	2.024 (0.488-8.392)	0.331
Aspirin/Clopidogre	1.086 (0.425-2.772)	0.863
Beta-blocker	1.442 (0.766-2.715)	0.257
ACEI	1.021 (0.140-7.434)	0.984
Statin	0.870 (0.310-2.450)	0.794
FAI ≥ -70.1	3.740 (2.012-6.953)	0.000
CT-FFR ≤ 0.80	6.842 (3.412-13.72)	0.000
SIS ≥ 4.5	3.910 (2.086-7.331)	0.000

ACEI - angiotensin converting enzyme inhibitor; BMI - body mass index; CAD - coronary artery disease; CT-FFR - CT-derived fractional flow reserve; CI - confidence interval; FAI - fat attenuation index; HR - hazard ratio; MACE - major adverse cardiac events; SIS - segment involvement score

**Table 3:** Multivariable Cox models for MACE in suspect CAD patients without SMuRFs

	Model 1		Model 2		Model 3		Model 4	

	HR (95% CI)	*P*-value	HR (95% CI)	*P*-value	HR (95% CI)	*P*-value	HR (95% CI)	*P*-value
Sex	1.903 (0.988-3.667)	0.054	1.496 (0.756-2.960)	0.247	1.533 (0.787-2.986)	0.209	1.243 (0.629-2.459)	0.531
SIS= 4.5	3.309 (1.727-6.342)	0.000	3.130 (1.617-6.060)	0.001	1.419 (0.670-3.003)	0.360	1.317 (0.621-2.793)	0.472
FAI = -70.1			3.011 (1.585-5.722)	0.001			3.300 (1.750-6.224)	0.000
CT-FFR= 0.80					5.010 (2.195-11.43)	0.000	5.375 (2.381-12.13)	0.000

CAD - coronary artery disease; CT-FFR - CT-derived fractional flow reserve; CI - confidence interval; FAI - fat attenuation index; HR - hazard ratio; MACE - major adverse cardiac events; SIS - segment involvement score.

**Table 4:** Incremental prognostic value of CT-FFR or FAI over SIS

	C-statistic	P-value	Absolute IDI	IDI P-value
Model 1: Sex + SIS ≥ 4.5	0.712			
Model 2: model 1 + FAI ≥ -70.1	0.739	0.169 (vs. model 1)	0.012	0.179 (vs. model 1)
Model 3: model 1 + CT-FFR ≤ 0.80	0.742	0.159 (vs. model 1)	0.006	0.261 (vs. model 1)
Model 4: model 1 + FAI ≥ -70.1+ CT-FFR ≤ 0.80	0.806	0.032 (vs. model 1)	0.070	< 0.001 (vs. model 1)

CT-FFR - CT-derived fractional flow reserve; FAI - fat attenuation index; IDI - integrated discrimination index; SIS - segment involvement score

## Discussion

The present study investigated the added prognostic value of FAI and CT-FFR beyond plaque burden in suspected CAD patients without SMuRFs. We showed that neither FAI nor CT-FFR added additional prognostic value over SIS. However, combining FAI and CT-FFR provided incremental prognostic value and improved cardiovascular risk stratification.

SIS, by providing a comprehensive measure of plaque burden, is increasingly becoming an important prognostic indicator. CT-FFR and FAI are feasible and non-invasive functional testing techniques and have been proposed as tools for assessing hemodynamically stenosis and vessel inflammation. The prognostic performance of SIS, CT-FFR, or FAI as a single indicator is well described. Rosendael et al.^20^ analysed 3547 suspected CAD patients and showed the prognostic value of SIS. It is well known that CT-FFR-guided treatment and revascularization can reduce the rate of MACE^21^,^22^. In a study of 492 patients who underwent CCTA and SPECT-MPI, FAI independently predicted MACE^14^. In line with previous studies, the present study revealed that FAI, CT-FFR or SIS was associated with MACE in suspected CAD patients without SMuRFs. Recent evidence suggests that CAD patients without SMuRFs have similar plaque progression rates as those with SMuRFs^23^. Our result is consistent with the hypothesis that non-traditional risk factor such as activated inflammation, hemodynamic stress, or plaque burden exacerbates the cardiovascular risk.

However, risk stratification based on single variable is an oversimplification of CAD pathophysiology. Considering this, several studies have evaluated the incremental prognostic value of variables to further risk stratification and guidance management of patients. In a study of 956 patients with suspected CAD, Ahmed et al. found that adding SIS to CCTA and SPECT significantly improved model prediction of MACE^9^. Nadjiri et al. showed SIS provided incremental prognostic value to models with calcium scoring^24^. A more recent study found that age-adjusted SIS significantly added risk reclassification to models with obstructive CAD^25^. Although SIS had incremental prognostic value beyond CCTA, SPECT, or calcium scoring, SIS solely reflected the anatomical characteristics of plaque and provided limited information on risk prediction in suspected CAD patients. Most recently, functional assessment has been well recognized. A study has focused on the importance of inflammation in atherosclerosis, even in no SMuRFs^26^. Likewise, the added predictive performance of CT-FFR has been examined. Hoshino et al.^27^ reported that the addition of FAI improved the discrimination ability for predicting MACE with FFR (FFR < 0.75). Bengs et al.^14^ found that FAI did not have added prognostic value beyond multimodality MPI including calcium scoring. Another study reported that CT-FFR ≥ 0.80 was a better predictor of MACE than severe CCTA (≥70% stenosis)^28^.

Our study showed how FAI and CT-FFR gradually increased risk stratification. To our knowledge, no prior studies have focused on the added prognostic role of FAI and CT-FFR over SIS. According to the present findings, we demonstrated that the single indicator of FAI or CT-FFR did not provide incremental prognostic value over SIS, suggesting that only one biomarker cannot offer sufficient prognostic information in this specific class of suspected CAD patients without SMuRFs. However, the combination of FAI and CT-FFR provided superior predictive performance. Our findings support the use of a multimarket strategy for risk prediction in suspected CAD patients without SMuRFs. These results indicate that the multimarket strategy of combining traditional CCTA scores and molecular biomarkers may provide complementary information to plaque development and have great potential to further improve disease prediction.

Our findings have potential clinical implications. First, these biomarkers can be obtained from CCTA images without additional scanning. Second, we focus on a unique cohort of suspected CAD patients without SMuRFs, highlighting the importance of considering CAD even in individuals without traditional risk factors. Third, our findings show the importance of hemodynamic stress and activated inflammation in the prognosis of CAD patients without SMuRFs. These biomarkers may represent potential treatment targets for MACE in patients without standard modifiable risk factors.

## Study limitations

Our study possessed several limitations. First, the study was carried out at a single center with a small sample size. In addition, the retrospective nature may result in selection bias. Second, the follow-up period was relatively short and longer periods of follow-up will be necessary.

## Conclusion

In suspected CAD patients without SMuRFs, neither FAI nor CT-FFR independently added incremental prognostic value over plaque burden. Combining FAI and CT-FFR had incremental prognostic value and improved cardiovascular risk stratification.
